# Photodegradation of the *Mycobacterium ulcerans* Toxin, Mycolactones: Considerations for Handling and Storage

**DOI:** 10.1371/journal.pone.0033600

**Published:** 2012-04-13

**Authors:** Estelle Marion, Soizic Prado, Camille Cano, Jérémie Babonneau, Sarah Ghamrawi, Laurent Marsollier

**Affiliations:** 1 Unité Inserm U892, Équipe 15, Nantes-Angers, France; 2 Groupe d'Etude des Interactions Hôte Pathogène, Université et CHU d'Angers, Angers, France; 3 Museum National d'Histoire Naturelle de Paris, Paris, France; Institut de Pharmacologie et de Biologie Structurale, France

## Abstract

**Background:**

Mycolactones are toxins secreted by *M. ulcerans*, the etiological agent of Buruli ulcer. These toxins, which are the main virulence factors of the bacilli, are responsible for skin lesions. Considering their specificity for *M. ulcerans* and their presence in skin lesions even at early stages, mycolactones are promising candidates for the development of a diagnostic tool for *M. ulcerans* infection. Stability of purified mycolactones towards light and heat has not yet been investigated, despite the importance of such parameters in the selection of strategies for a diagnosis tool development. In this context, the effects of UV, light and temperature on mycolactone stability and biological activity were studied.

**Methodology/Principal Findings:**

To investigate the effect of these physical parameters, mycolactones were exposed to different wavelengths in several solvents and temperatures. Structural changes and biological activity were monitored. Whilst high temperature had no effect on mycolactones, UV irradiation (UV-A, UV-B and UV-C) and sunlight exposure caused a considerable degradation, as revealed by LC-MS and NMR analysis, correlated with a loss of biological activity. Moreover, effect of UVs on mycolactone caused a photodegradation rather than a phototransformation due to the identification of degradation product.

**Conclusion/Significance:**

This study demonstrates the high sensitivity of mycolactones to UVs as such it defines instructions for storage and handling.

## Introduction

*Mycobacterium ulcerans* is the causative agent of Buruli ulcer. The disease, being the third most common mycobacterial infection after tuberculosis and leprosy, is considered as an emerging or re-emerging tropical disease. The incidence of Buruli ulcer is gradually increasing with the majority of cases occuring in poor rural communities [1]–[3]. Most cases are diagnosed in Africa, few being reported in Asia, Australia and South America. *M. ulcerans* causes extensive destruction of the skin and soft tissues with the formation of large ulcers, as well as bone and joint lesions. Without treatment, the lengthy healing process generally results in deep scaring and severe functional disabilities [4]–[5]. The painless and feverless progression explains why the treatment is frequently sought too late. Notably, about 25% of those infected – particularly children – become permanently disabled and endure social, economic and developmental problems.

Skin lesions are caused by a family of toxins called mycolactones, which are the main virulence factors of *M. ulcerans* [6]–[8]. Mycolactones present two original characteristics: they are the only known mycobacterial toxins, and are macrolide lipids, a rarity among bacterial toxins that are usually proteic or peptidic. These polyketide-derived macrolides are composed of a lactone core with two unsaturated side chains and are responsible for the yellow color of *M. ulcerans* colonies. The first description of mycolactones was that of two *cis/trans* isomers A/B extracted and purified from *M. ulcerans* culture [6]. Both isomers are produced by the majority of strains involved in human disease. Other mycolactones were isolated from *M. ulcerans* (Mycolactones C and D) [9] and from other mycobacterial species pathogenic to fish (*M. marinum*, mycolactone F) [10] and batrachians (*M. liflandii*, mycolactone E) [11]. However, genomic analysis suggests that all mycobacteria producing mycolactones can be considered as a single specie [12]. The genes encoding the six enzymes involved in toxin production are located on a giant plasmid [8]. Following synthesis, the toxin is secreted in bacterial membrane-derived vesicles and concentrated in the extracellular matrix, which acts as a reservoir [13]. Although mycolactones are known for their cytotoxic activity and immuno-modulation effects, the molecular mechanisms of their activities are still unknown [7], [14]–[15].

Primary diagnosis is usually based on clinical findings, but confirmatory methods require specialized laboratories for: direct smear examination, culture of *M. ulcerans*, polymerase chain reaction (PCR) and histopathological analysis [2]. Unfortunately, no simple diagnostic tool is currently available for rural areas of developing countries. This is of particular importance since, with the introduction of antibiotic treatment in 2004, the early uncomplicated stages of Buruli ulcer can be easily treated locally by a combination of rifampin and an aminoglycoside for eight weeks associated, if necessary, with simple excision under local anesthesia [16].

Mycolactones have been recently detected in tissues from patients with early stage of Buruli ulcer [17]. The toxins, being specific to *M. ulcerans*, would be a promising marker to develop a new diagnostic test. Nevertheless, their small size and lipid nature hinder the development of tools for their detection. For this purpose, knowledge about toxin stability has to be improved. Indeed, sensitivity of several other polyketides (such as aflatoxin B1, a naturally occurring fungal mycotoxin, or erythromycin, a macrolide antibiotic) to light and temperature has already been established [18]–[20]. Previous studies have also suggested that non purified *M. ulcerans* toxin was heat-stable [21]–[22]. In the light of such findings, several questions about stability of mycolactones have been raised. Therefore, we investigated the mycolactone response to light and heat. Our results demonstrated that heat (100°C) had no effect on mycolactones. Conversely, exposure to UVs or sunlight resulted in their rapid degradation correlated with a loss of biological activity.

## Materials and Methods

### Ethics statement

Ethics statement was not required: No human participant was involved and animal work was not conducted in this study.

### Mycolactone extraction and purification

Mycolactones A/B were purified from *M. ulcerans* extracts as previously described [6]. Briefly, *M. ulcerans*, African strain S4018 isolated from a patient in Benin, was grown in Middlebrook 7H10 agar supplemented with Oleic Albumin Dextrose Catalase growth supplement. Bacteria were re-suspended in chloroform-methanol (2∶1, v/v) and cell debris were removed after centrifugation. Folch extraction was realized by adding 0.2 volume water. The organic phase was dried and phospholipids were precipitated with ice-cold acetone. The acetone-soluble lipids were loaded on a thin layer chromatography plate and eluted with chloroform-methanol-water (90∶10∶1) solvent as mobile phase. The yellow band with a retention factor of 0.23 was scraped, filtered, evaporated and then resuspended in absolute ethanol. The concentration was determined by measuring the absorbance (λ_max_ = 362 nm, logε = 4.29), and purity was evaluated by High Performance Liquid Chromatography (HPLC).

**Table 1:** Main spectra of light source.

Light source	Main spectra (nm)
UV tube 254	254
UV tube 312	312
UV tube 365	365
Incandescent lamp	390–760
Fluorescent lamp	390–760
Sunlight	280–800
Red light	500–3000
Dark	NA*

* NA: not applicable.

### Detection and quantification of mycolactones by HPLC

HPLC was performed with a C18 column (Zorbax Eclipse XDB-C18, 9,4×250 mm), and mycolactones were eluted by a 33-min gradient from 90 to 10% water for phase A and 10 to 90% acetonitrile for phase B, with a flow rate of 1 ml/min. Chromatogram was monitored at 363 nm and an external standard was used to quantify the toxin. Detection limit of mycolactones was established at 7 ng.

### Mycolactone exposure to UV, light and temperature

- **Effect of temperature and light.** Mycolactones A/B were diluted to 10 µg/ml in acetonitrile and stored at −20°C as aliquots in glass tubes until use. Some aliquots were heat-treated at temperatures ranging from 60°C to 100°C during 1 or 6 h. Some aliquots were placed in quartz tubes at 3 different wavelengths: 254 nm, 312 nm and 365 nm to study the effect of UVs. Other aliquots were exposed to different irradiations in sealed standard laboratory glass tubes to mimic laboratory conditions for storage and manipulation, and exposed to UVs as mentioned above, direct incandescent light, neon light or red light. Various exposure times were tested: 5 min, 15 min, 30 min, 45 min, 1 h, 2 h, 6 h and 12 h. Mycolactones were also subjected to direct sunlight exposure in sealed glass tubes during 5 min, 15 min, 30 min, 45 min, 1 h, 2 h and 6 h. In parallel to each exposure condition, controls were placed in dark for equivalent period of time and stored similarly. Following treatments, mycolactones were stored at −20°C in amber glass tubes before analysis. All experiments were repeated 3 times.
- **Effect of UV in different solvants.** Mycolactones were diluted to 100 µg/ml in ethanol and acetone, aliquoted in glass tubes and exposed to UV at 312 nm. Mycolactones were also diluted to 100 µg/ml in acetonitrile : some glass tube aliquots were dried while others were placed directly in amber tubes prior exposure to UV at 312 nm or light.

### Mass Spectrometry (MS) and Nuclear Magnetic Resonance (NMR) analysis

Mass spectra were recorded on an API Q-STAR PULSAR (Applied Biosystem).^13^C NMR spectra were recorded on an AC 300 BRUKER spectrometer operating at 75.47 MHz (for ^13^C and ^1^H) and 2D-NMR spectra on an Avance 400 BRUKER spectrometer operating at 400.13 MHz. For heteronuclear multiple bond correlation (HMBC) experiments, the delay (1/2*J*) was 150 ms.

### LC-MS analysis

LC-MS experiments were done on an Ultimate 3000 nanoHPLC system (Dionex) connected to an Agilent 1100 UV detector and an ESI-QqTOF (QStar® Pulsar i, Applied Biosystems®). The separation was achieved on an ACE3-C18® column (3 µm×5 cm×0.5 mm, ACE Scotland).The elution gradient was 10%–90% water-acetonitrile to 100% acetonitrile over 46 min at a flow rate of 40 µl/min. LC/MS analyses were carried out in positive electrospray ionization mode. 5 µl of extract (14 µg/ml, ethanol solvent) were injected in the column.

### Biological activity of mycolactones

The functional integrity of toxins was assessed based on their cytotoxic activity. Macrophages, Raw 264.7, were cultured in RPMI 1640 supplemented with 10% heat-inactivated fetal calf serum. Tests were performed in 384-well plates by seeding 5000 cells in 50 µl culture medium. After overnight incubation at 37°C, the medium was replaced by fresh medium containing mycolactones at various concentrations. After an incubation of 24 h, the percentage of cell mortality was quantified with Vialight Plus kit (Lonza). Samples were tested in triplicates. All toxin samples in acetonitrile were diluted 100 times in RPMI just before the assay. RPMI medium containing 1% acetonitrile was not cytotoxic and was used in control assays.

### Statistical analysis

Differences between treatments were tested for statistical significance using Kruskal-Wallis and Wilcoxon tests.

**Figure 1:**
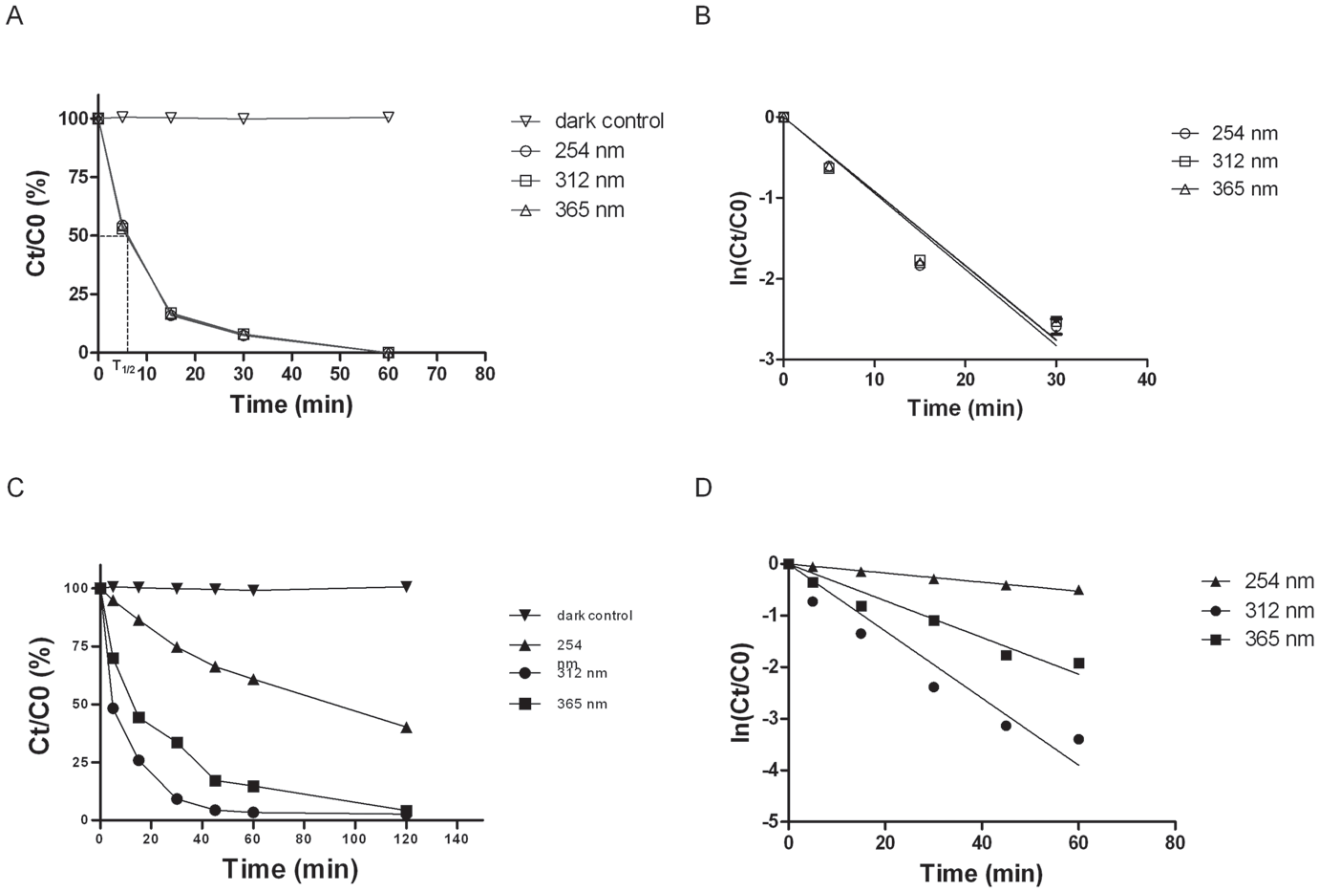
Variation in the quantity of native mycolactones after exposure to UV irradiation in quartz and glass tubes. (A) Remaining native mycolactones after different exposure times and UV wavelengths in quartz tubes. (B) Representation of the linear relationship between mycolactones and time irradiation to calculate half-life time in quartz tubes. (C) Remaining native mycolactones after different exposure times and UV wavelengths in glass tubes. (D) Representation of the linear relationship between mycolactones and time irradiation to calculate half-life time in glass tubes. Results in (A) and (C) represent mean values of triplicate experiments. Standard deviations represent less than 2.5% of the obtained mean values. C0 and Ct are the concentration at times 0 and t, t is the irradiation time.

**Table 2:** Kinetic parameters of mycolactone degradation at different UV wavelengths.

Light source	Intensity (mW/cm2)	Ln(Ct/C0) quartz tube	T1/2 (min) quartz tube	Ln(Ct/C0) glass tube	T1/2 (min) glass tube
254 nm	3	−0,092t	7.5	−0.0088t	78.8
312 nm	3	−0,094t	7.4	−0.065t	10.7
365 nm	3	−0,092t	7.5	−0.035t	19.5

## Results

### Impact of light on mycolactone structure and biological activity

To study the impact of irradiation, mycolactones were exposed to three different UV wavelengths (254, 312 and 365 nm, corresponding respectively to UV-A, UV-B, UV-C), in quartz tubes or glass tubes. Moreover, two artificial white lights (fluorescent and incandescent lights), one red light and direct sunlight exposures were applied to mycolactones in glass tubes. The light sources and their main spectra are listed in table 1. The stability of mycolactones in different solvents was also evaluated. After exposure to light, the quantity of remaining native toxins was determined by HPLC and their cytotoxic effect was evaluated.

#### (i) Effect of UV

Exposing mycolactone in quartz tubes to 3 different UV wavelengths caused a rapid decrease in the quantity of native mycolactones (figure 1A). A linear relationship between ln(Ct/C0) and irradiation time indicated that the degradation followed a first-order kinetics (C0 and Ct are concentrations at times 0 and t, t is the irradiation time) with a half-life time calculated at 7.5 min (figure 1B and table 2). To place in laboratory storage and manipulation, we studied the effect of UVs on mycolactone stored in glass tubes that may limit the UV diffusion. In glass tubes, for each wavelength, the quantity of mycolactone decreased slightly less than in quartz tubes (*p*<0,001). Moreover, as shown in figure 1D, mycolactone decrease kinetic is different for the 3 wavelengths, with calculated half-life times of 10.7, 19.5 and 78.8 min for 312, 365 and 254 nm, respectively (Table 2). No native toxins were detected after 6 h for 312 and 365 nm and 12 h for 254 nm irradiation. Control toxins were stable at room temperature in the dark. Interestingly, we observed that mycolactones exposed to UV for 6 hours lose their yellow color (figure S1) suggesting a structure modification or a degradation.

**Figure 2:**
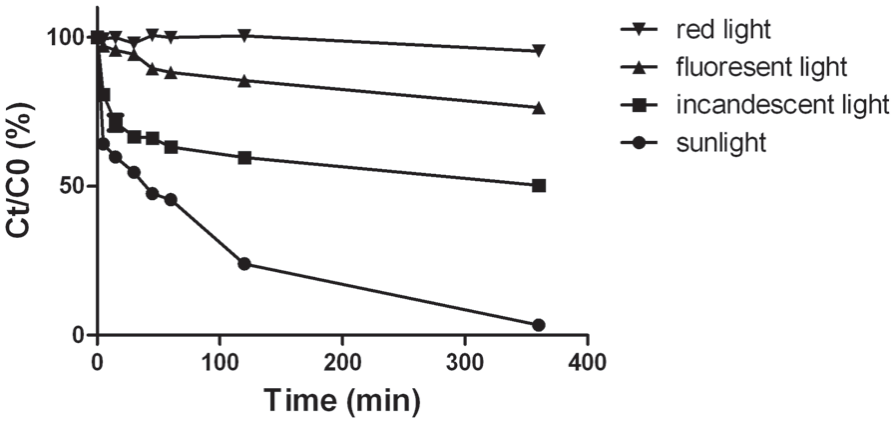
Photodegradation of mycolactones after exposure to natural and artificial lights. Results represent mean values of triplicate experiments. Standard deviations represent less than 2.5% of the obtained mean values. C0 and Ct are the concentration at times 0 and t, t is the irradiation time.

#### (ii) Effect of artificial lights and sunlight

Exposure of mycolactones (in glass tubes) to sunlight led to an important decrease in native toxin quantity. Fifty percent of the toxins were lost after 30 min of exposure (figure 2) and after 6 h, only 3% of mycolactones remained. The incandescence light caused a slower decrease in native toxins, since about 50% of the toxins were detected after 6 h. The neon light had lesser effects with 70% of the toxins unchanged after 6 h. In contrast, there was no variation in the quantity of toxins after exposure to red light, as compared to the control. Since the remaining mycolactones was always higher than 50% with the 2 artificial lights, the half-life times could not be determined. Likewise, half-life time could not be defined for sunlight because irradiation intensity varies during the day. These results suggested a phototransformation or photodegradation after exposure to light.

**Figure 3:**
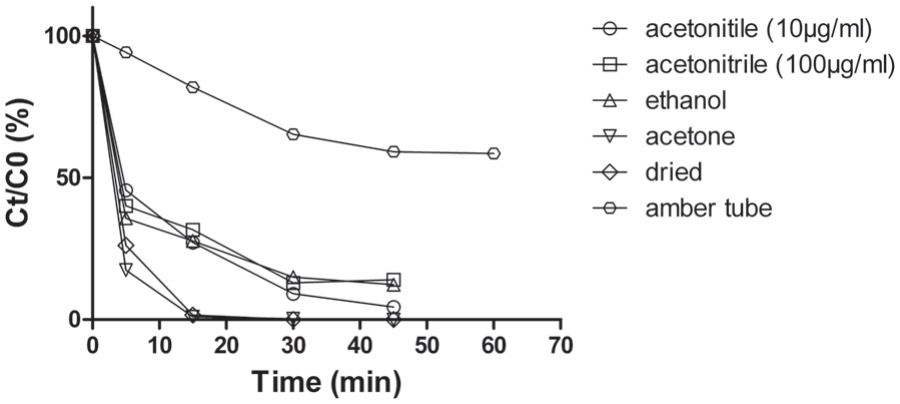
Variation in the quantity of native mycolactones after exposure to UV at 312 nm in different solvents and in amber tubes. Results represent mean values of triplicate experiments. Standard deviations represent less than 2.5% of the obtained mean values. C0 and Ct are the concentration at times 0 and t, t is the irradiation time.

#### (iii) Effect of solvents

To evaluate the impact of the solvent on mycolactone stability exposed to UV, a similar experiment was performed on dried mycolactone or solubilized in acetone or ethanol. However, to quantify samples by HPLC, mycolactones had to be diluted in acetonitrile. So, for this experiment,the initial quantity of mycolactones was 10 times concentrated (100 µg/ml). As the kinetic of degradation of mycolactones was not dependent on the initial concentration of toxin (figure 3), results from different conditions could be compared. Interestingly, while ethanol did not alter the kinetic of degradation of the mycolactone to UV (figure 3), acetone impaired the stability of mycolactone when exposed to UV. Similarly, dried mycolactones were less stable than mycolactones placed in acetonitrile or ethanol (figure 3). Storage of mycolactones in amber glass tubes partially protected against UV since 95% of mycolactone remained after 5 minutes versus 45% in acetonitrile. Moreover, no degradation was recorded for mycolactones stored in amber tubes exposed to natural light (data not shown).

**Figure 4:**
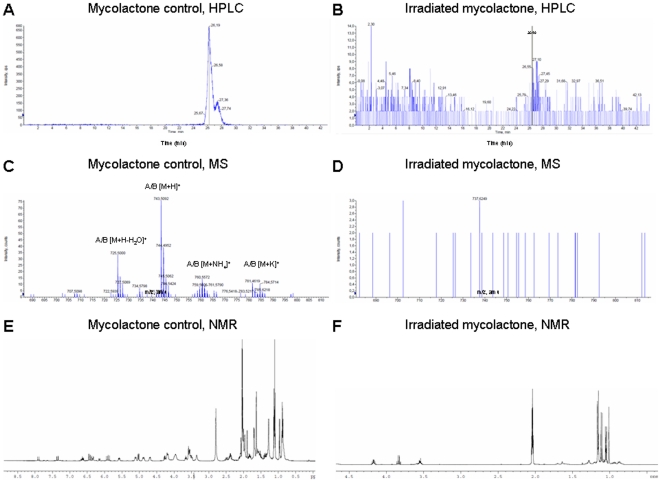
LC-MS and NMR analysis of mycolactones after 12-h UV irradiation. (A) Extracted ion current chromatogram for the mycolactone control shows a 26.19-min retention time and (B) for the mycolactones after UV exposure. (C) Positive ESI mass spectrum at 26.19 min revealing the presence of mycolactones A/B [M+H]+ at *m/z* : 743.5092 for the control and (D) its absence UV exposure. (E) ^1^H NMR spectrum (Acetone-d6, 298 K, 400 MHz) of mycolactone control and (F) mycolactones after exposure.

**Figure 5:**
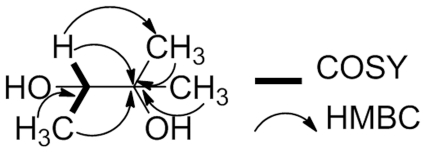
Key ^1^H-^1^H COSY (COrrelation SpectroscopY) and ^1^H-^13^C HMBC (Heteronuclear Multiple-Bond Correlation) correlations for one fragment.

#### (iv) LC-MS and NMR analysis of mycolactones

Extracted ion current chromatogram for control mycolactones A/B (i.e. 12 hours in the dark) [M+H]^+^ at *m/z* : 743.5 from a LC-MS run of the native mycolactones revealed a maximum at a retention time (rt) of 26.19 min (figure 4A). The mass spectrum obtained at this retention time presented the characteristic pattern of ionization of mycolactones A/B (figure 4C). Nevertheless, extracted ion of mycolactone sample following 12 hour-irradiation pointed out the absence of a signal, suggesting its complete degradation (figure 4B). This correlated with a low intensity of the most abundant peaks (intensity 14 cps) (figure 4B). The mass spectrum at rt 26.19 min of irradiated mycolactones displayed no ion peak characteristic of mycolactones A/B, confirming its absence (figure 4D). Noteworthy, LC-MS analysis of sample of mycolactones following 12 h of irradiation suggested a complete decomposition into small chemical entities difficult to detect by MS (figure 4B and D).

**Figure 6:**
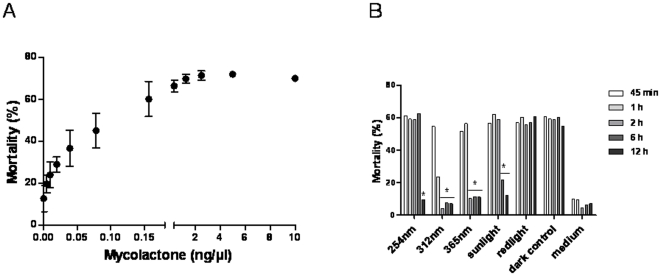
Cytotoxic evaluation of mycolactones after irradiation. (A) Cytotoxic effect of mycolactones: a dose response. Raw 264.7 cells were incubated with mycolactones during 24 hours. (B) Cytotoxic effect evaluation of mycolactones after light and UV exposure. Medium is a culture medium containing 1% of acetonitrile. Asterisks indicate significant (p<0.05) effect of exposure to light on mycolactone cytotoxic effect.

This observation was consistent with the ^1^H NMR profile of irradiated mycolactones. Indeed, our sample of mycolactones gave ^1^H NMR ((CD_3_)_2_CO) data identical with the one reported by Gunawardana et *al*. [23] (Figure 4E). On the other hand, the ^1^H NMR spectrum of irradiated mycolactones presented only 3 methine signals (δ_H_ 4.18, 3.82 and 3.60) as well as 5 methyl groups (two methyl doublets at δ_H_ 1.17 and 1.00 and three methyl singulets at δ_H_ 1.15, 1.10 and 1.07) (figure 4F). These were consistent with the ^13^C-*J* modulated NMR (CD_3_)_2_CO) spectrum (figure S2) which exhibited resonance corresponding to only 10 carbons including two quaternary, three oxymethine and five methyl groups. Similarly, no ester carbonyl or olefinic carbons were detected. Extensive 2D NMR analysis confirmed the degradation of the molecule and suggested the presence of three distinct fragments. Nevertheless, these data allowed us to propose a structure for only one of them (figure 5). Finally, at shorter UV exposure times (5 min, 15 min, 1 h), no by-products were identified, suggesting that UV irradiation caused a degradation of mycolactones and not a phototransformation (data not shown).

#### (v) Evaluation of mycolactone cytotoxicity after irradiation

The influence of mycolactone concentration on cell mortality was first investigated. Cells were incubated during 24 hours with mycolactones before performing the analysis. After logarithmic transformation, a linear relationship was observed between mycolactone concentration and cytotoxic effect on cells between toxin concentrations of 0.004 and 0.15 ng/µl (figure 6A and figure S3). Mortality of the cells reached a plateau for a concentration of mycolactones of 0.3 ng/µl. The cytotoxic effect was then evaluated for samples exposed to different light sources, at 45 min, 1, 2, 6 and 12 hours. The results showed that the decrease in the quantity of mycolactones after irradiation with UV wavelengths or sunlight correlated with the loss of cytotoxic activity (figure 6B). In contrast, the mycolactones subjected to red light exhibited the same mortality effect on cells compared to mycolactones stored in the dark. These results demonstrated that UV exposure results in a marked decrease in biological activities of mycolactones correlated with photodegradation.

### Mycolactone structure and cytotoxicity preserved after heating

Mycolactones were submitted to temperatures ranging from 60 to 100°C during 1 or 6 h. The native mycolactone concentration was measured in each condition by HPLC and no variation was recorded. This was in agreement with the preservation of the cytotoxic effect of the toxin after exposure to elevated temperature. Likewise LC-MS and NMR analysis revealed no structural changes in the toxins (Data not shown).

## Discussion

Mycolactones, which are toxins specifically produced by *M. ulcerans*, the causative agent of Buruli ulcer, are responsible for skin lesions characteristic of the disease. Currently, for case confirmation of *M. ulcerans*, World Health Organization recommends performing at least two of the following tests: PCR, direct smear examination, culture, and histology. PCR is the most sensitive and specific technique, but it is not adapted to endemic areas since it requires specific equipments, expensive reagents and skilled personnel. Except for few endemic countries, PCR confirmation is performed in European countries, complicating patient management. Recognizing the importance of laboratory confirmation of cases, it is now recommended that countries should ensure that at least 50% of all cases are tested by PCR. One of the priorities of WHO is the development of a new diagnostic tool, adapted to endemic areas, simple, cost and time effective, and specific to *M. ulcerans* infection. Mycolactones A/B, which are specific to *M. ulcerans* and present around sites of infection, are therefore promising targets for the development of such a test.

In this study all experiments were performed in acetonitrile, ethanol or acetone or with dried mycolactones since toxin solubility in aqueous medium is low. Our results showed that mycolactones are not sensitive to elevation of temperature. On the contrary, mycolactones are highly sensitive to wavelengths corresponding to UV-C (254 nm), UV-B (312 nm) and UV-A (365 nm). Mycolactones are also sensitive to sunlight exposure and, to a lesser extent, to artificial lights (fluorescent and incandescent), but not to red light, which emits wavelengths higher than 500 nm. UVs reaching the earth are UV-A (98%) and UV-B (2%) and represent less than 5% of the wavelengths of the sun. Emission of wavelength in artificial lights is mainly in visible spectra (400 nm–700 nm), but some UV-A are also present in low amount, explaining the degradation of mycolactones after several hours exposed to these lamps. Following irradiation of mycolactones with redlight, no degradation of the toxins occurred even after several hours, confirming that mycolactones is only sensitive to low wavelengths (UVs).

Mycolactones are polyketides with a lactone function belonging to the family of macrolides. In literature, several molecules with a lactone function were described to be photosensitive and thermosensitive [18]–[19], [24]. For instance, photodegradation was described for Aflotoxin B1, a toxin from *Aspergillus flavus*, contaminating food, especially crops like peanuts [18]. UV irradiation caused a phototransformation of the toxin for which a pathway has been proposed. UV irradiation is therefore used as a physical method to abolish biological activities of this teratogen toxin. In the case of mycolactones, no pathway were proposed because no by-products were identified, indicating a photodegradation.

Phototherapy is sometimes used in human skin diseases, like psoriasis or eczema, but rarely in infectious diseases. Currently, some dermatophytes (fungi) are susceptible to UV treatment both *in vitro* and *in vivo*, so the use of such treatment in clinical practises is discussed [25]. In the case of *Propionibacterium acnes*, a Gram positive bacterium, causing acne vulgaris, blue light phototherapy seems to be beneficial in the treatment of the disease [26]. Although mycolactones are light sensitive, phototherapy might not to be an additional treatment of Buruli ulcer, since in closed lesions or undermined edges of ulcerative stage, mycolactones are not subjected to light.

Nevertheless, these results are essential to establish the best conditions of handling and storage of mycolactones in laboratory. Together with the absence of native mycolactone in LC-MS and NMR analysis after irradiation, and the marked decrease in biological activity of the toxins, our results formally demonstrate photodegradation of mycolactones. Therefore, guidelines could be proposed for handling mycolactones in order to develop a diagnostic tool. To avoid photodegradation, clinical samples and mycolactone extracts should be kept in the dark, but variations of temperature of storage does not seen to be important.

## Supporting Information

Figure S1 **Left tube contains mycolactone in acetonitrile at a concentration of 1 mg/ml.** After 6 hours exposure to UVs, the yellow colour disappears (right tube) indicating that mycolactone structure is modified by UVs.
(TIF)

Figure S2 **^13^C-** ***J*** **modulated spectrum NMR ((CD_3_)_2_CO, 75.47 MHz, 298 K).**
(TIF)

Figure S3 **Logarithmic transformation indicating a linear relationship between mycolactone concentration (x) and cytotoxic effect on cells (y) for concentrations ranging from 0.004 to 0.15 ng/µl.**
(TIF)
