# Supplementary figures and images for: Photodegradation of the Mycobacterium ulcerans Toxin, Mycolactones: Considerations for Handling and Storage
Source: PLoS One. 2012 Apr 13;7(4):e33600. doi: 10.1371/journal.pone.0033600 (PMC3326021; doi:10.1371/journal.pone.0033600)

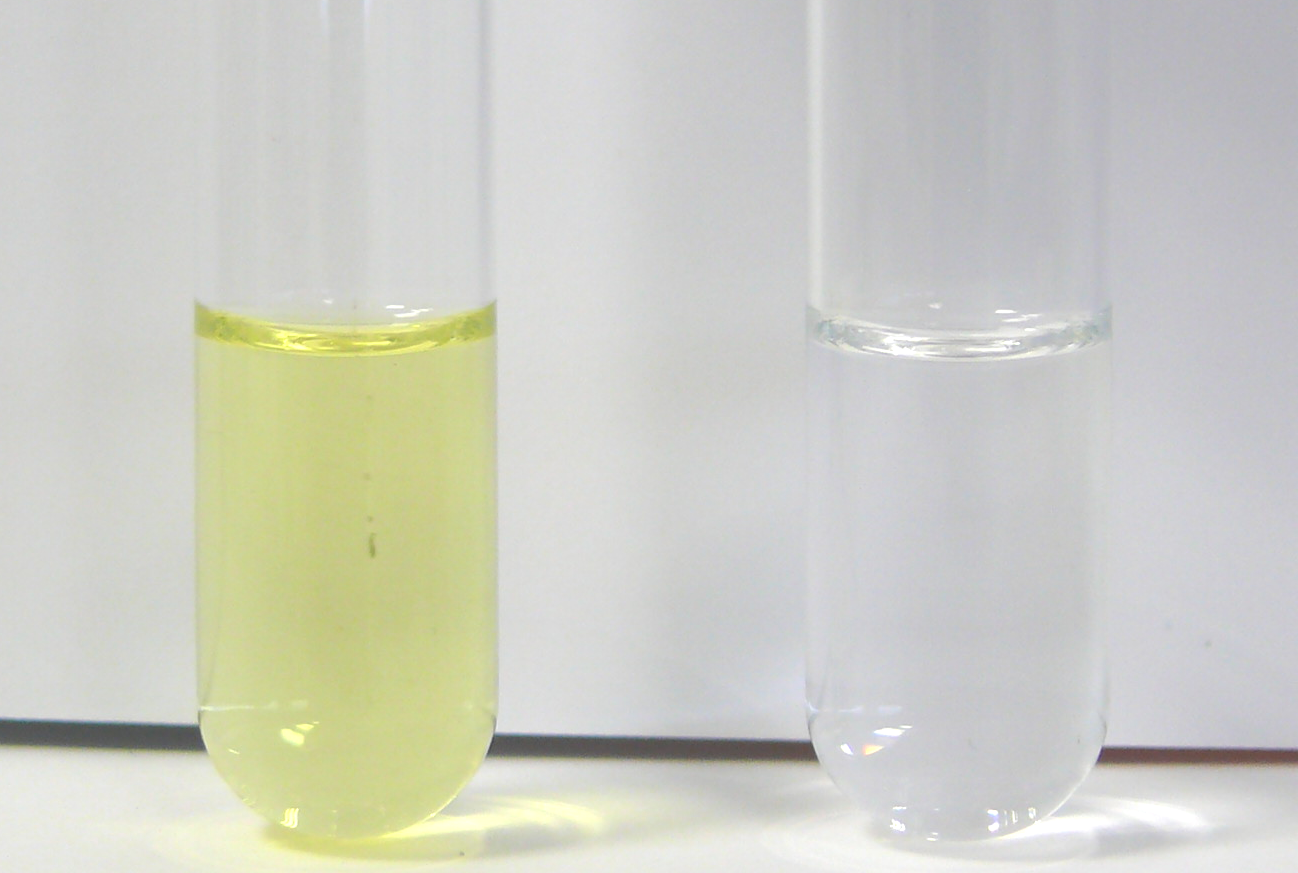

Supplement: Figure S1 — Left tube contains mycolactone in acetonitrile at a concentration of 1 mg/ml. After 6 hours exposure to UVs, the yellow colour disappears (right tube) indicating that mycolactone structure is modified by UVs. (TIF) [file pone.0033600.s001.tif]

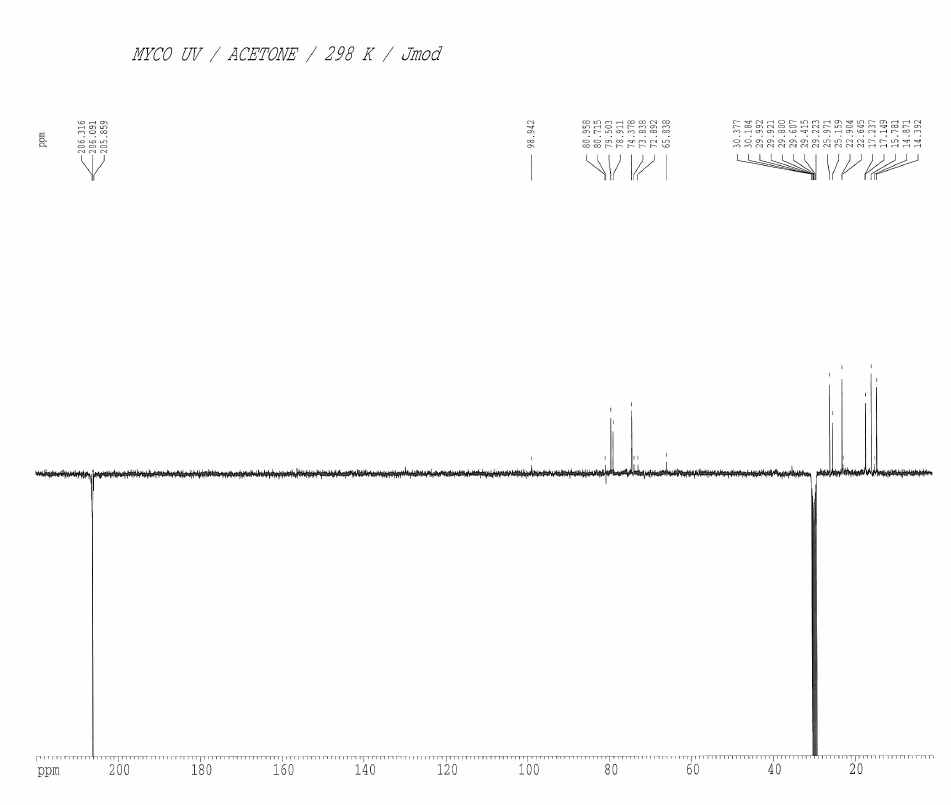

Supplement: Figure S2 — 13C- J modulated spectrum NMR ((CD3)2CO, 75.47 MHz, 298 K). (TIF) [file pone.0033600.s002.tif]

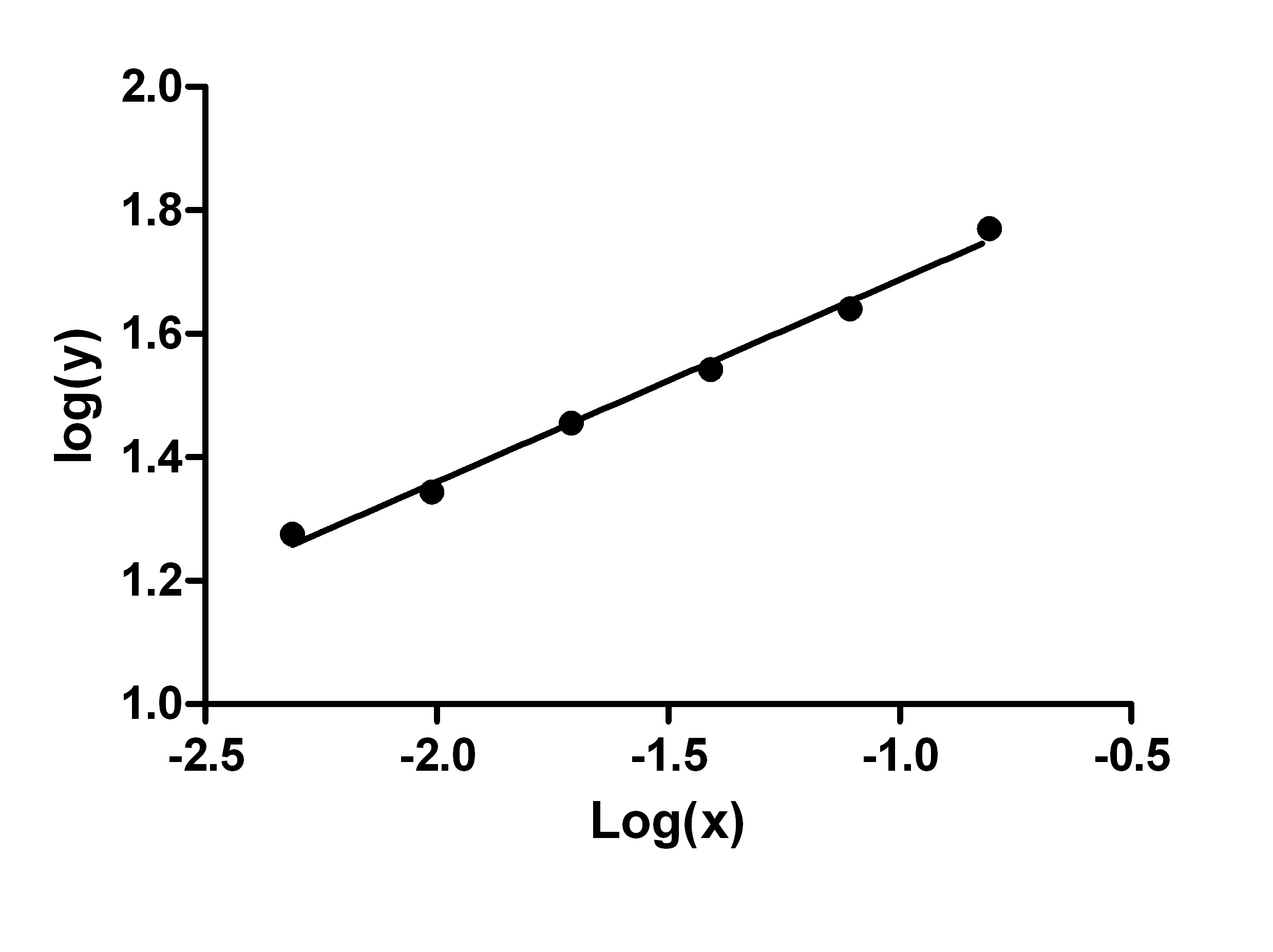

Supplement: Figure S3 — Logarithmic transformation indicating a linear relationship between mycolactone concentration (x) and cytotoxic effect on cells (y) for concentrations ranging from 0.004 to 0.15 ng/µl. (TIF) [file pone.0033600.s003.tif]
